# Case Report: DOCK8 Deficiency Without Hyper-IgE in a Child With a Large Deletion

**DOI:** 10.3389/fped.2021.635322

**Published:** 2021-06-14

**Authors:** Edna Venegas-Montoya, Aidé Tamara Staines-Boone, Luz María Sánchez-Sánchez, Jorge Alberto García-Campos, Rubén Antonio Córdova-Gurrola, Yuridia Salazar-Galvez, David Múzquiz-Zermeño, María Edith González-Serrano, Saul O. Lugo Reyes

**Affiliations:** ^1^Immunology Service, Hospital de Especialidades Unidad Medica de Alta Especialidad (UMAE) 25 del Instituto Mexicano del Seguro Social (IMSS), Monterrey, Mexico; ^2^Pediatrics Service, Hospital de Especialidades Unidad Medica de Alta Especialidad (UMAE) 25 del Instituto Mexicano del Seguro Social (IMSS), Monterrey, Mexico; ^3^Infectious Disease Department, Hospital de Especialidades Unidad Medica de Alta Especialidad (UMAE) 25 del Instituto Mexicano del Seguro Social (IMSS), Monterrey, Mexico; ^4^Pediatrics Service, General Hospital 1, Saltillo, Mexico; ^5^Immunodeficiencies Lab, National Institute of Pediatrics, Mexico City, Mexico

**Keywords:** DOCK8 deficiency, large deletion, Hyper-IgE, case report, combined immune deficiency, literature review

## Abstract

Autosomal recessive (AR) DOCK8 deficiency is a well-known actinopathy, a combined primary immune deficiency with impaired actin polymerization that results in altered cell mobility and immune synapse. DOCK8-deficient patients present early in life with eczema, viral cutaneous infections, chronic mucocutaneous candidiasis, bacterial pneumonia, and abscesses, together with eosinophilia, thrombocytosis, lymphopenia, and variable dysgammaglobulinemia that usually includes Hyper-IgE. In fact, before its genetic etiology was known, patients were described as having a form of Hyper-IgE syndrome, a name now deprecated in favor of genetic defects. We describe a school-age male patient with a clinical picture suggestive of DOCK8 deficiency, except for high serum IgE or a family history: early onset, failure to thrive, eczema, warts, condyloma, bronchiolitis, pneumonia, recurrent otitis media, bronchiectasis, candidiasis, leukocytosis, eosinophilia, high IgA, low IgG, and low CD4+ T cells. We were able to confirm the diagnosis through protein expression and whole-exome sequencing. We review the clinical, laboratory, and genetic features of 200 DOCK8-deficient patients; at least 4 other patients have had no elevated IgE, and about 40% do not have Hyper-IgE (above 1,000 IU/mL). Despite this, the constellation of signs, symptoms, and findings allow the suspicion of DOCK8 deficiency and other actinopathies.

## Introduction

An estimated 7,000 individually rare diseases together afflict about 1 in every 17 humans (1). Individuals with rare diseases endure a diagnostic odyssey in which they consult an average of more than seven physicians for more than 6 years before someone suspects their correct diagnosis (2). Diagnostic errors are an important source of waste, complaints, complications, and deaths (3). Inborn errors of immunity (IEIs) are a group of congenital rare diseases with increased susceptibility to infection, autoimmunity, inflammation, allergy, and cancer (4). Combined immune deficiencies (CIDs), which stunt the numbers or responses of lymphocytes, are among the most severe IEIs (5); they manifest themselves since very early in life with adverse reactions to live vaccines, severe eczema, chronic diarrhea, atopic and/or bleeding diatheses, failure to thrive and opportunistic infections, as well as a life-long risk of autoimmune disease and malignancy.

Actinopathies are CIDs that prevent the polymerization of actin, thus impairing cell mobility and the immune synapse of hematopoietic cells (6). Autosomal recessive (AR) DOCK8 deficiency is one of the better-known actinopathies. The dedicator of cytokinesis 8 is a large protein that activates the small GTPase CDC42, which is essential for the reorganization of actin (7). DOCK8 also interacts with the WIP-WASp complex, regulates STAT3, and promotes a Th17 CD4+ differentiation (8, 9). The defective actin accumulates in natural killer (NK) cells and cripple their cytotoxicity function (10). Although DOCK8-deficient patients were first described as suffering from a type of Hyper-IgE syndrome (11), it soon became clear that this is a distinct entity of graver prognosis (12). Here, we describe the case of a DOCK8-deficient school-age boy with normal IgE serum levels, who nevertheless had other signs and findings that allowed suspecting the diagnosis.

## Case Report

**Figure 1:**
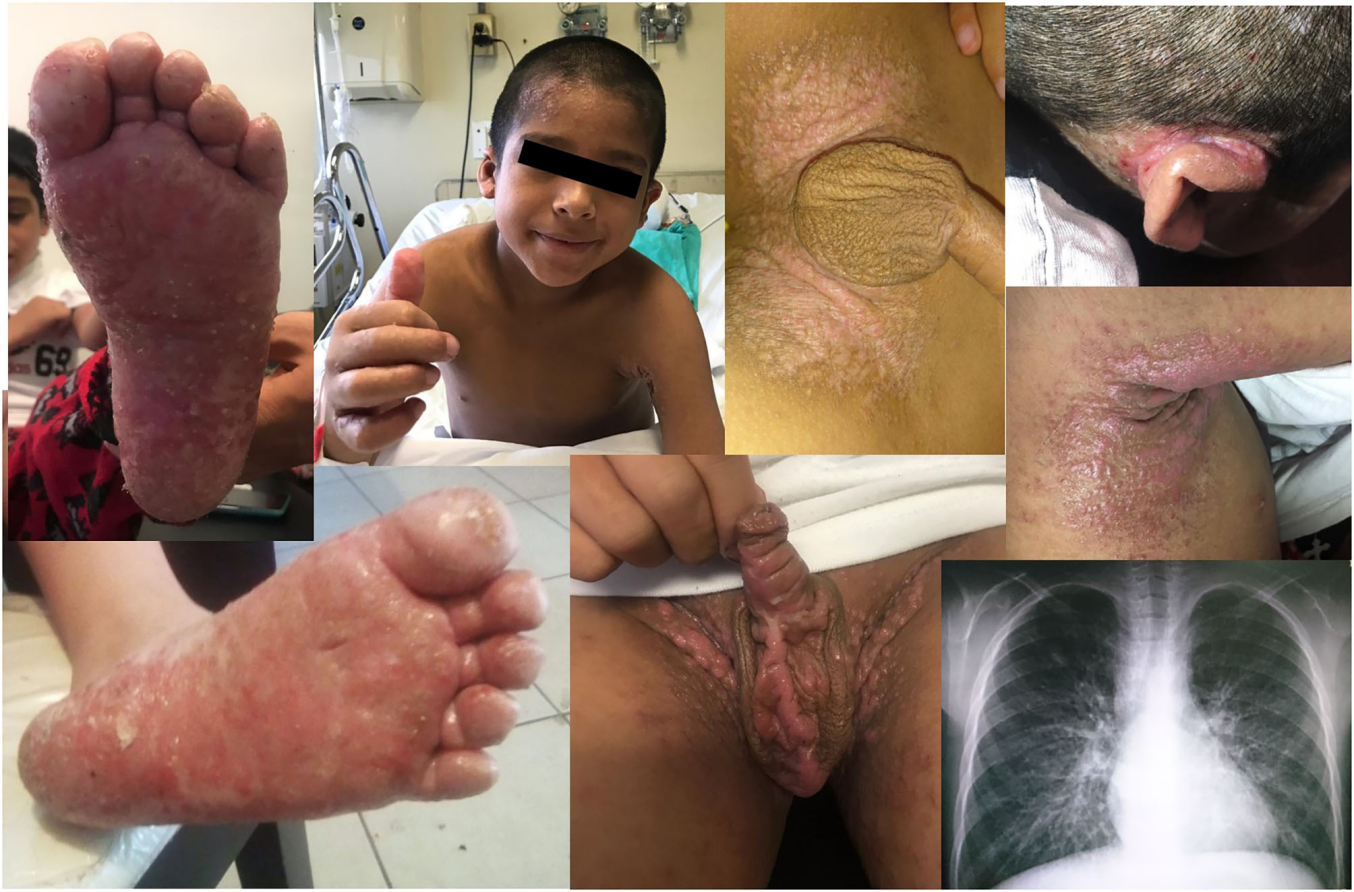
Some clinical features of DOCK8 deficiency. Plain warts and discolored skin patches in forehead, trunk, hands, and soles. Severe retroauricular and axillar eczema. Lower right corner: chest radiograph showing bilateral bronchiectases and perihilar interstitial infiltrates.

A 6-year-old male patient was referred to our care for a history of eczema, respiratory infections, warts, and mucocutaneous candidiasis. Born from a non-consanguineous family in Northern Mexico, he tolerated the *Bacille Calmette-Guérin* (BCG) vaccine shortly after birth, and his family history was significant only for two spontaneous abortions. He started at age 1 month old with extensive eczema attributed to cow's milk protein allergy. Later, the patient also developed one episode of bronchiolitis, three of pneumonia, and three of otitis media with effusion, as well as twice-a-month intermittent diarrhea, disseminated flat warts, chronic mucocutaneous candidiasis, and condyloma acuminatum around the scrotum and groin folds (see Figure 1).

On physical examination, low weight, short stature, absent tonsils, a dry skin with extensive eczema, and mild hepatosplenomegaly were noted. Plain warts and discolored skin patches were corroborated in the forehead, trunk, hands, and soles, as well as mycotic skin lesions in feet and genitals.

**Figure 2:**
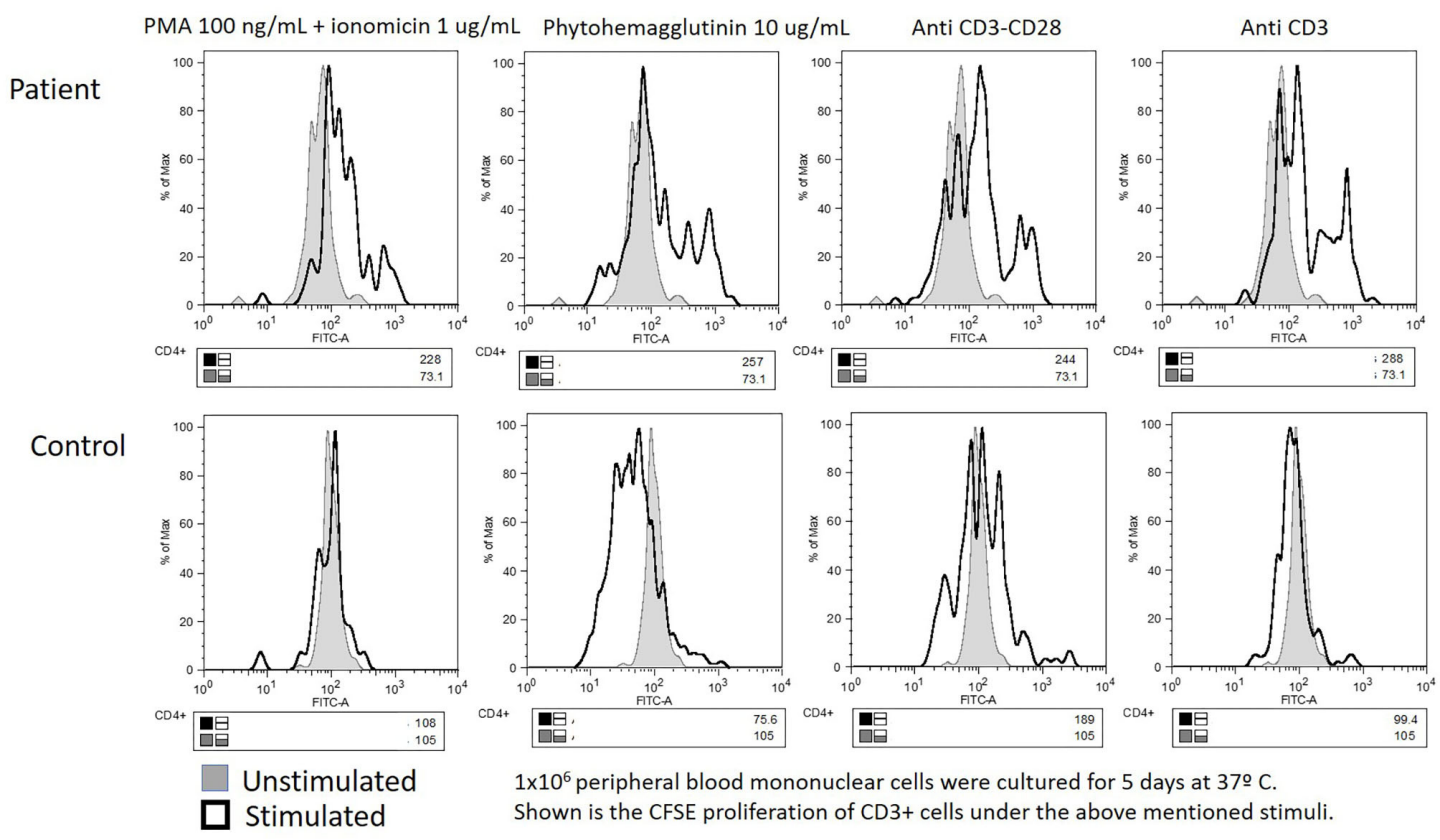
Equivocal carboxyfluorescein succinimidyl ester lymphoproliferation assay: impaired in both the patient's and the “travel control” blood samples.

High-resolution CT of the chest revealed bilateral bronchiectases. Blood counts reported leukocytosis (26,500–73,400 cells/mm^3^) and hyper-eosinophilia (14,900–30,000–75,000/mm^3^), with Hb 12.7 g/dL, neutrophils 9,570, lymphocytes 4,890, monocytes 1,520, and 252,000/mm^3^ platelets. Serum immunoglobulin G (IgG) was low at 408 mg/dL, with high IgA (550) and normal IgM (66 mg/dL); total serum IgE was marginally high at 95 IU/mL. CD4+ T cells were also low at 365 cells (33%), with the rest of lymphocyte subsets within the normal range (CD3+ 1,669, CD8+ 1,070, CD19+ 574, CD16/56+ 226 cells/mm^3^). Serologies for HIV, EBV, and CMV were all negative, as was a skin prick allergy test. The mitogen-induced carboxyfluorescein (CFSE) lymphoproliferation assay was equivocal: both the patient and a healthy (travel) control showed a limited response (see Figure 2).

A provisional diagnosis of CID was given. Alternative diagnoses we entertained for this patient were as follows: Wiskott–Aldrich syndrome (WAS), HIV/AIDS (CD4+ lymphopenia), and leukemia (hypereosinophilia). However, a bone marrow aspirate and a CGL myeloid mutation panel were reported as normal. Despite an almost normal serum IgE, DOCK8 deficiency was included in the differential diagnosis, considering the constellation of early onset, failure to thrive, eczema, warts, condyloma, bronchiolitis, pneumonia, recurrent otitis media, bronchiectasis, candidiasis, leukocytosis, eosinophilia, high IgA, low IgG, and low CD4+ lymphocytes.

**Figure 3:**
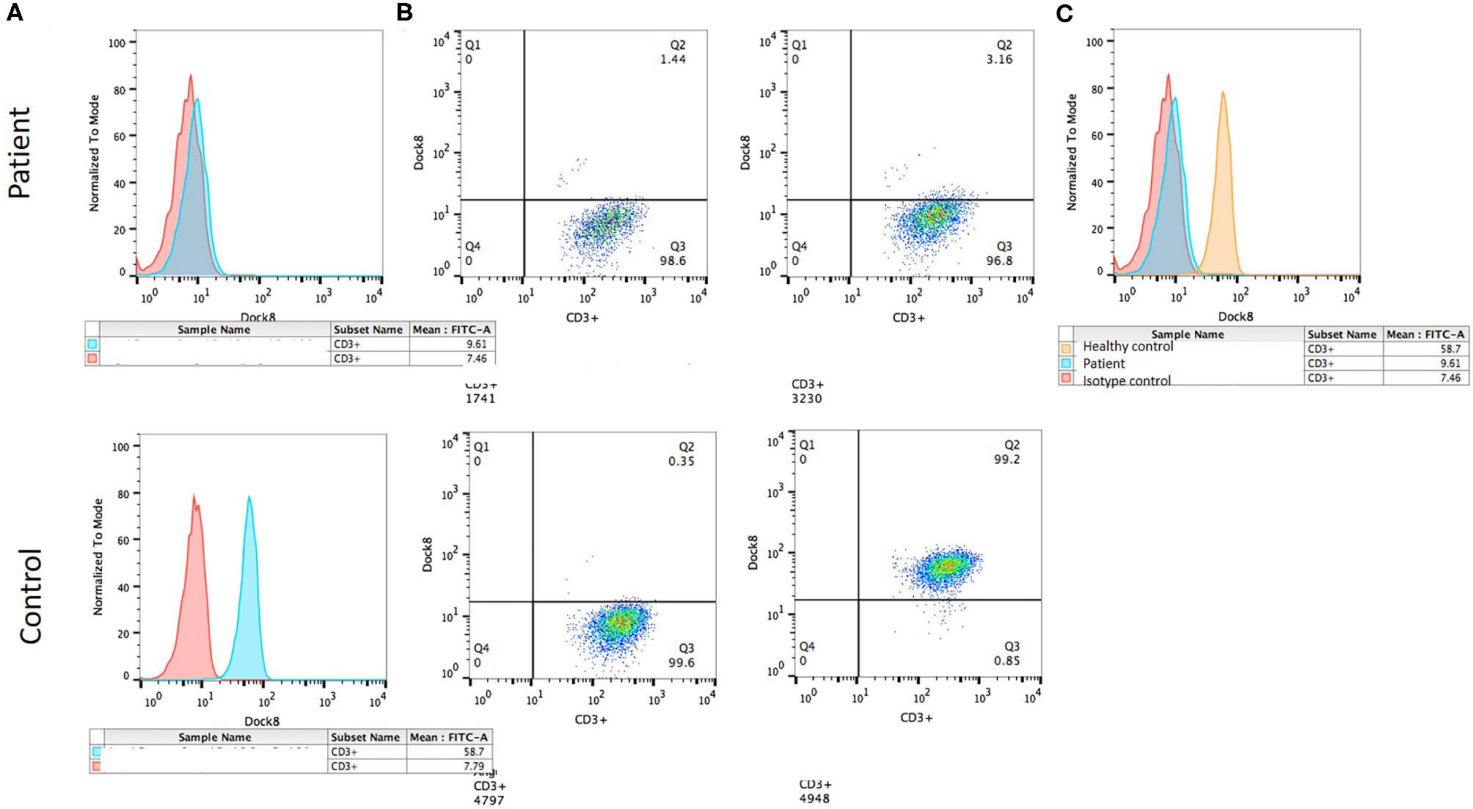
DOCK8 expression by flow cytometry. **(A)** Histograms showing mean fluorescence intensity: isotype control (red) and DOCK8 expression (blue) in the patient (above) and the healthy control (below). **(B)** Dot plot showing DOCK8 expression in CD3+ cells in the patient (above) and the control (below). **(C)** Histogram comparing DOCK8 expression in the healthy control (orange) and the patient (blue).

**Figure 4:**
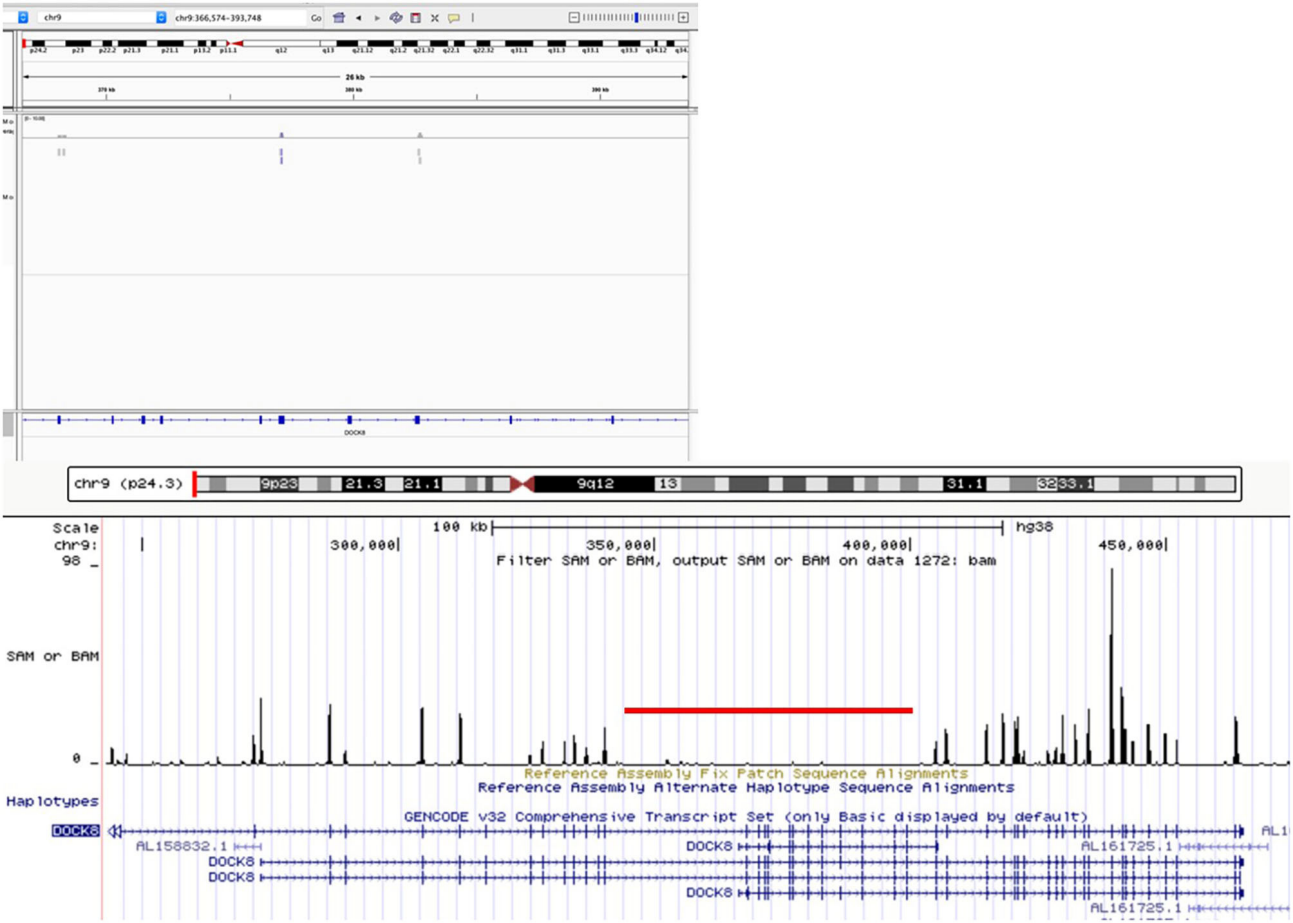
Whole-exome sequencing visualization on Integrative Genomics Viewer (IGV) and UCSC Genome browser, showing a large homozygous deletion of *DOCK8* spanning introns 14 through 26, more than 50 kb.

Flow cytometry expression for WASp was slightly reduced in peripheral blood mononuclear cells, whereas DOCK8 expression was null (see Figure 3). Through whole exome sequencing (WES), we identified a large homozygous deletion that spans from introns 14 to 26, ~60,000 base pairs (Figure 4). The patient is currently stable under treatment with oral prophylactic antibiotics (TMP/SMZ and itraconazole), together with monthly intravenous immunoglobulin; he is awaiting hematopoietic stem-cell transplantation (HSCT).

## Discussion

We describe a school-age male patient with an early onset of eczema, respiratory, and cutaneous infections caused by bacteria, viruses, and fungi. He developed hepatosplenomegaly, failure to thrive and bronchiectasis, hypereosinophilia, CD4+ lymphopenia, and dysgammaglobulinemia. Despite him not having Hyper-IgE, a high index of suspicion for DOCK8 deficiency led to a genetic diagnosis at age 7. DOCK8 expression was null, and a large genomic deletion, spanning introns 14 through 26, was identified through WES. Although this is a single atypical case, we were able to confirm the molecular (no protein expression) and genetic (large genomic deletion) diagnoses, while the patient awaits HSCT.

The differential diagnosis of suspected DOCK8 deficiency includes the Wiskott–Aldrich (eczema, respiratory infections, susceptibility to bacteria and viruses, high serum IgE), Loeys–Dietz (food allergies, arterial aneurysms, high IgE), complete DiGeorge (combined deficiency, mucocutaneous candidiasis, atopy), Comel–Netherton (brittle hair, eczema, eosinophilia, high IgE), IPEX (severe eczema, neonatal onset, eosinophilia, diarrhea, high IgE), and Omenn (alopecia, erythema, severe eczema, lymphopenia, eosinophilia, high IgE) syndromes; ARPC1B deficiency (thrombocytosis, eosinophilia, high IgG, IgA, and/or IgE), IRAK4/MyD88 deficiency (early onset of recurrent invasive bacterial infections, impaired inflammatory response + dysgammaglobulinemia/high IgE), CARD11 negative dominance (combined immune deficiency + atopy + high IgE), STAT3 negative dominance (eczema, cutaneous abscesses, chronic mucocutaneous candidiasis, high IgE), and its phenocopy ZNF341 deficiency; PGM3 (early onset eczema, rhinitis, asthma, recurrent bacterial, fungal and viral infections, bronchiectasis, skin vasculitis, lymphopenia, hypergammaglobulinemia, high IgE) and IL6ST (atopic dermatitis, food allergy, recurrent respiratory infections, eosinophilia, high IgE) deficiencies; MST1 (STK4) deficiency (warts, recurrent bacterial, viral and fungal infections, abscesses, autoimmunity, CD4+ lymphopenia); IL2RB deficiency (eczema, food allergies, combined deficiency with autoimmunity, lymphadenopathy, and hypergammaglobulinemia); and GATA2 haploinsufficiency (cutaneous viral infections, susceptibility to mycobacteria and fungi, bone marrow failure/myelodysplastic syndrome/leukemia, low B cells).

To date, more than 250 DOCK8-deficient patients have been described, 98% of which had high serum IgE (13, 14). At least four patients have been noted not to have elevated IgE, whereas almost 40% do not qualify as Hyper-IgE, defined as total serum IgE of more than 1,000 IU/mL. Most patients, however, have some degree of elevated serum IgE (i.e., >60 IU/mL), and about 95% have peripheral blood eosinophilia, together with some combination of eczema, food allergy, sinopulmonary bacterial, and cutaneous viral infections. It is thus the constellation of signs of symptoms, the clinical and laboratory *gestalt*, which supports the diagnostic suspicion of DOCK8 deficiency in the absence of Hyper-IgE, as in this case. Our patient had up to 56,680 eosinophils per mm^3^ at one point.

In 2016, Kienzler et al. described a DOCK8-deficient female patient without high serum IgE, who had compound heterozygous variants that resulted in a hypomorphic expression (15). Furthermore, they found a somatic reversion in T cells to which they attributed a much milder phenotype (no severe infection or atopy), despite having low CD4+ cells, low IgM and IgG, and impaired vaccine responses. Among the 136 patients included in Aydin et al.'s study (13), only 3 had serum IgE levels within the normal range (while in a dozen more, IgE was high without being “hyper”: below 1,000 IU/mL); all 3 had large genomic deletions and severe phenotypes. Patients with autosomal dominant (AD) STAT3 loss of function (LOF) without Hyper-IgE levels have also been reported (16).

The Hyper-IgE syndrome was first called “Job syndrome” and was then renamed when IgE was discovered and found to be markedly high in the serum of the originally described patients (17), whose phenotype was inherited in an AD fashion. Eventually, a group of autosomal recessive patients with a marginally similar phenotype (i.e., they shared a high serum IgE, eosinophilia, abscesses, and skin lesions) was characterized (11), and their genetic etiologies were identified between 2009 and 2018 (18–23). DOCK8 deficiency is classified, and better understood, as a combined immune defect; its prognosis and treatment are quite different from those of STAT3 loss of function. One common pitfall in our field is to think that all the so-called Hyper-IgE syndromes are the same, just with different etiologies; another important one is to rule out or accept the provisional diagnosis based on the misnomer “Hyper-IgE syndrome,” that is, to expect the total IgE serum levels to be “through the roof.”

More than once (16), the convenience of calling inborn errors of immunity diseases by their genetic defect and the need to abandon misleading or erroneous names such as Hyper-IgE and Hyper-IgM syndromes have been put forward (24). Our case report is yet another example. Apart from insisting in this change, we would also like to recall the most prevalent clinical, laboratory, and genetic features of DOCK8-deficient patients (see Tables 1–3): eczema, food allergy, molluscum/plain warts, abscesses, candidiasis, pneumonia, Hyper-IgE (98%), eosinophilia (96%), low IgM, and low T cells. Patients with DOCK8 deficiency have a life-long cumulative risk of developing aneurysm, cancer, and fatal infections, which is why the recommended treatment, the only one with curative potential, is HSCT.

**Table 1:** Clinical features reported in 200 patients with DOCK8 deficiency.

**Feature**	**Number of patients affected**		**%**
Viral cutaneous: (warts, molluscum)	Molluscum	61/165	36.9%
	Warts 59/164	35.9%
	Herpes simplex virus	90/167	53.8%
	Varicella Zoster Virus	36/167	21.5%
Eczema		193/197	97.9%
Candidiasis		117/145	80.6%
Abscess		118/193	61.1%
Pneumonia		79/139	56.8%
Food allergy		123/153	80.3%
Asthma		69/153	45%
Autoimmunity		19/194	9.7%
Malignancy		28/198	14.4%
Bronchiectasis		74/178	41.5%
Aneurism		9/69	13%

**Table 2:** Laboratory features found in 200 patients with DOCK8 deficiency.

**Feature**	**Engelhardt et al. (14)**	**Aydin et al. (13)**
Thrombocytosis	37.5% (24/64)	NR
Eosinophilia	91.5% (54/59)	96%
Lymphopenia	18.9% (11/58)	20%
Low IgM	62% (36/58)	64%
Low IgG	3.4% (2/58)	64%
Low CD4	28.5 % (16/56)	45%
Low CD8	29% (16/55)	38%
Low CD19	5.4% (3/55)	12%
Low CD16/56	26% (13/50)	28%
High IgE	98.3% (61/62)	98%
IgE > 2,000	38.7% 24/62 (IgE > 10,000)	NR

**Table 3:** Genetic variants identified in 60 patients with DOCK8 deficiency.

**Variant type**	**Proportion**
Multi-exon deletions	27 (45%)
Nonsense	6 (10%)
Missense	0
Splice-site	12 (20%)
Single-exon deletion	8 (13.3%)
Small indels	4 (6.6%)
Others	3 (5%)

To conclude, patients with DOCK8 deficiency may not have high total serum IgE levels, yet the astute clinician will maintain the diagnosis in his/her differential based on (any combination of) early onset eczema, food allergy, unusual infection, abscess, autoimmunity, eosinophilia, dysgammaglobulinemia, and CD3+ lymphopenia. “Hyper-IgE syndrome” is an incorrect name for several quite different diseases, from a time before their true nature was known, and we should abandon the misnomer in favor of more descriptive diagnoses and genetic etiologies.

## Data Availability Statement

The original contributions presented in the study are included in the article/supplementary material, further inquiries can be directed to the corresponding author/s.

## Ethics Statement

The studies involving human participants were reviewed and approved by Research and Ethics Committee, National Institute of Pediatrics. Written informed consent to participate in this study was provided by the participants' legal guardian/next of kin. Written informed consent was obtained from the minor(s)' legal guardian/next of kin for the publication of any potentially identifiable images or data included in this article.

## Author Contributions

EV-M and AS-B cared for the patient, directed the diagnostic approach, wrote the manuscript, and reviewed the final version. LS-S, DM-Z, JG-C, RC-G, and YS-G cared for the patient and reviewed the final version of the manuscript. MG-S performed and analyzed the CFSE lymphoproliferation and protein expression by flow cytometry assays and reviewed the final version. SL processed and analyzed the whole-exome sequencing and conceived and wrote the manuscript. All authors contributed to the article and approved the submitted version.

## Conflict of Interest

The authors declare that the research was conducted in the absence of any commercial or financial relationships that could be construed as a potential conflict of interest.

## Abbreviations

AIDS: Acquired immune deficiency syndrome
AR: autosomal recessive
AD: autosomal dominant
BCG: Bacille Calmette-Guérin
DOCK8: Dedicator of cytokinesis 8
EBV: Epstein-Barr virus
HIV: human immunodeficiency virus
HSCT: hematopoietic stem-cell transplantation
IEI: inborn errors of immunity
IMSS: Mexican Institute of Social Security
INP: National Institute of Pediatrics
DNA: deoxyribonucleic acid
NK: natural killer.
